# Case Report: Metagenomic Next-Generation Sequencing for Diagnosis of Human Encephalitis and Endophthalmitis Caused by Pseudorabies Virus

**DOI:** 10.3389/fmed.2021.753988

**Published:** 2022-01-14

**Authors:** Weiqian Yan, Zhiping Hu, Yingchi Zhang, Xiaomei Wu, Hainan Zhang

**Affiliations:** Department of Neurology, Second Xiangya Hospital, Central South University, Changsha, China

**Keywords:** pseudorabies virus, Suid herpesvirus 1, endophthalmitis, next-generation sequencing, encephalitis

## Abstract

**Purpose:**

The objective of our study was to report a case of encephalitis and endophthalmitis caused by pseudorabies virus (PRV), identified using metagenomic next-generation sequencing (mNGS).

**Case Presentation:**

A 54-year-old worker, from a swine slaughterhouse, developed signs of severe encephalitis, including fever, disturbance of consciousness, hypopnea, and status epilepticus, after finger injury at work. The PRV sequences were successfully identified from the blood, cerebrospinal fluid (CSF), and aqueous humor of the patient through mNGS, which was further verified using a Sanger sequencing.

**Conclusion:**

Our case emphasizes the importance of mNGS in early diagnoses of infectious diseases, and gives a clue that PRV can spread across species and infect human. It is necessary to carry out a skin protection and education about disease prevention for people who have close contact with swine.

## Introduction

Pseudorabies virus (PRV), also called Aujeszky disease virus or Suid herpesvirus 1, belongs to Alphaherpesvirinae subfamily of the family Herpesviridae and is widely distributed around the world. Swine are the main natural hosts and sources of PRV infections (1). The PRV is latent in the peripheral nervous systems (PNS) of infected swine, which shed large quantities of virus in bodily secretions and excretions. The PRV is mainly spread *via* direct contact, and may also be transmitted by water, air, and contaminated fomites (2). There are some reports on human PRV infection based on clinical manifestations and epidemiological history. However, few researches had conclusive evidences for human PRV infection due to the rigorous conditions of viral PCR and viral culture and low accuracy of antigen-antibody test (3–5). In recent years, with the application of metagenomic next-generation sequencing (mNGS) in the diagnosis of infectious diseases, human encephalitis and/or endophthalmitis caused by PRV has been reported, confirming the possibility of cross-species infection of PRV (6–11). The PRV related infection has received increasing attention since the first case of human endophthalmitis caused by PRV was reported in 2018 (6). Given the close relationship between human and swine, we report this case to emphasize the importance of mNGS in early diagnosis and highlight the threat to public health.

## Case Presentation

**Figure 1:**
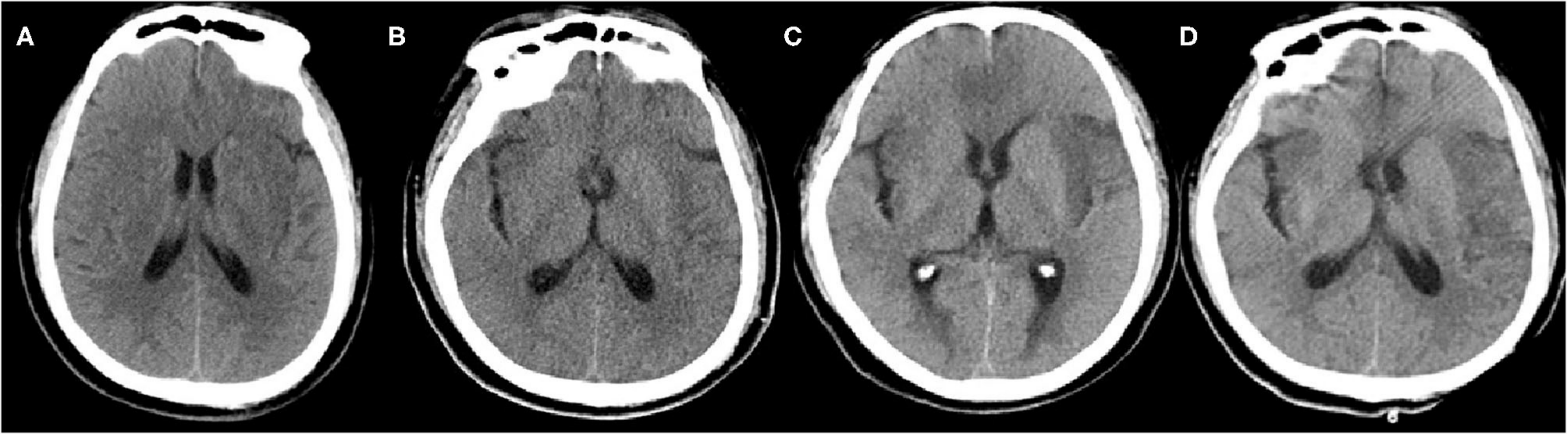
The plain CT brain imaging on the 8th day **(A)**, 15th day **(B)**, 19th day **(C)**, and 24th day **(D)** after initial symptoms.

A 54-year-old male, who worked as a butcher of the slaughterhouse in Hunan Province, China, was admitted to the Neurology Intensive Care Unit (NICU) of Second Xiangya Hospital, Central South University, due to recurrent fever for a duration of 8 days and convulsions with impaired consciousness for 3 days. He obtained some minor cuts on his left hand during the autopsy process of a dead swine 14 days before his initial symptom occurred. The initial symptoms (day 1) presented with cough and fever (the highest temperature of 39°C). No other neurological symptoms were presented at that time. He was then admitted to the local hospital and was diagnosed with upper respiratory tract infection, receiving an antibiotic treatment. However, his condition was not improving. On day 5, after initial symptoms, he developed fuzzy consciousness, headache, fecal, and urinary incontinence. Plain brain computer tomography (CT) was normal. Analysis of cerebrospinal fluid (CSF) showed white blood cells (WBC) of 4 × 10^6^/L (0–5 × 10^6^/L) and protein content of 0.63 g/L (0.15–0.45 g/l) (Table 1). The results of CSF further indicated that it was an early phase of viral meningitis. He was given an antiviral treatment (Acyclovir and Ribavirin) and antibiotic therapy (Piperacillin/tazobactam). On day 6, he developed facial and limb convulsions and was treated with anti-epileptic therapy (sodium valproate and midazolam). He was given an emergency orotracheal intubation and ventilator-assisted ventilation, due to shortness of breath. On day 8, after the initial symptoms, he was referred to our hospital due to status epilepticus and disturbance of consciousness. On admission, he presented with a body temperature of 39°C, heart rate of 107 bpm (beats per minute), blood pressure of 155/94 mmHg, and oxygen saturation (SpO_2_) of 99% at an inspired oxygen concentration of 30% with mechanical ventilation, and no spontaneous breaths were detected. Glasgow Coma Scale (GCS) was E1VTM1. Pupils were both 3 mm in diameter. The left pupil was sluggishly reactive to light and the reflex of the right one disappeared. Although he had neck stiffness and Kernig signs, long tract signs, such as hyperreflexia, Hoffman's signs, as well as Babinski signs were not detected. Some minor cuts were found on his left hand (Supplementary Figure 1). Laboratory findings were WBC 12.01 × 10^9^/L (3.5–9.5 × 10^9^/L), hemoglobin (Hb) 127 g/dl (115–150 g/dl), sodium 148.3 mmol/L (135–145 mmol/l), potassium 3.4 mmol/L (3.5–5.5 mmol/l), creatinine 89 μmol/L (62–115 μmol/L), albumin 39.7 g/L (40–55 g/l), alanine transaminase (ALT) 90.6 U/L (9–50 U/l), and aspartate transaminase (AST) 49.3 U/L (15–40 U/l). Serological tests for HIV, TP, hepatitis B/C, and TB (T-SPOT) were negative. The cytomegalovirus antibody IgG (CMV-IgG) of 19.300 IU/mL, herpes simplex virus type I antibody (HSV) IgG of 26.76 COI (positive), rubella virus antibody IgG (RU-IgG) of 20.800 IU/mL, and *Toxoplasma gondii* antibody IgG (TOX-IgG) of <0.130 IU/ml were found. The IgM against all pathogens was negative. Lumbar puncture indicated an opening pressure of 290 mmH_2_O (80–180 mmH_2_O), CSF showed WBC of 10 × 10^6^/L (0–5 × 10^6^ /L), protein of 0.37 g/L (0.15–0.45 g/L) and glucose of 3.05 mmol/L (synchronous blood glucose of 9.7 mmol/L) (Table 1). Besides, CSF latex agglutination test (LAT), microbiological tests including the Gram/acid-fast bacillus (AFB) were negative, as well as the autoimmune encephalitis antibodies including NMDAR, AMPA1, AMPA2, LGI1, CASPR2, GABABR, IgLON5, DPPX, GAD65, GlyR1, DRD2, mGluR5, mGluR1, and Neurexin-3a, which were tested based on a cell-based assay (Kingmed Diagnostics Co., Ltd.). On day 8, Plain CT brain imaging was still normal after initial symptoms (admission day) (Figure 1A). With the suspicion of viral meningitis on admission, he was treated with Acyclovir (500 mg, Q8h), 20% Mannitol (125 ml, Q8h), Glycerol fructose (250 ml, Q12h), Piperacillin/tazobactam (4.5 g, Q8h), fluid therapy and nutritional support. The epileptic seizures of the patient were difficult to bring under control, propofol was used intravenously to stop status epilepticus. Meanwhile, oral antiepileptic therapy including Levetiracetam and Oxcarbazepine were applied. On day 11, after initial symptoms, pathogen reads PRV was detected in CSF, blood, and aqueous humor, using PACEseq mNGS, so we accordingly added Phosphonoformate (3 g, Q8h) combined with Acyclovir (500 mg, Q8h) to enhance the antiviral treatment. However, the condition of the patient did not see any significant improvement. On day 15, after initial symptoms, Plain CT brain imaging showed patchy hypo-density in the bilateral frontotemporal lobe (Figure 1B). Methylprednisolone Sodium Succinate (120 mg, Qd) was intravenously given for 7 days, followed by oral prednisone (60 mg, Qd) treatment. There was no improvement of the consciousness of the patient. Even when all the sedatives used to control epileptic were stopped, he was still in deep coma. On day 19, CT brain imaging showed multiple patchy hypo-density in right basal ganglia, left thalamus, bilateral frontotemporal lobe, and bilateral insular lobe (Figure 1C). On day 24, the lesion site of CT brain is basically the same as before (Figure 1D), which indicated that the present treatment might not be effective. During hospitalization, the patient was unable to wean off mechanical ventilation, although the pulmonary infection had been basically healed. Given that the prognosis of the patient was extremely poor, the patient was voluntarily discharged to local hospital for palliative treatment.

**Table 1:** Dynamic CSF analysis over the course of hospitalization.

**CSF-Variable**	**Intracranial pressure (mmH_**2**_O)**	**WBC (×10** ^**6**^ **/L)**		**Protein (g/L)**	**Glucose (mmol/L)**	**Chloride (mmol/L)**	**CSF-Culture**
		**Differential count (%)**					
		**Monocyte**	**Coenocyte**				
5th day	/	4	0	0.63	normal	106.8	/
8th day	290	10	4	0.37	3.05	132.4	(–)
11th day	350	4	6	0.78	3.14	127	(–)
16th day	400	42	6	0.78	3.62	125.5	(–)
19th day	400	55	15	0.69	4.09	131.4	/
23th day	310	45	15	0.80	4.57	144	/

## mNGS

**Figure 2:**
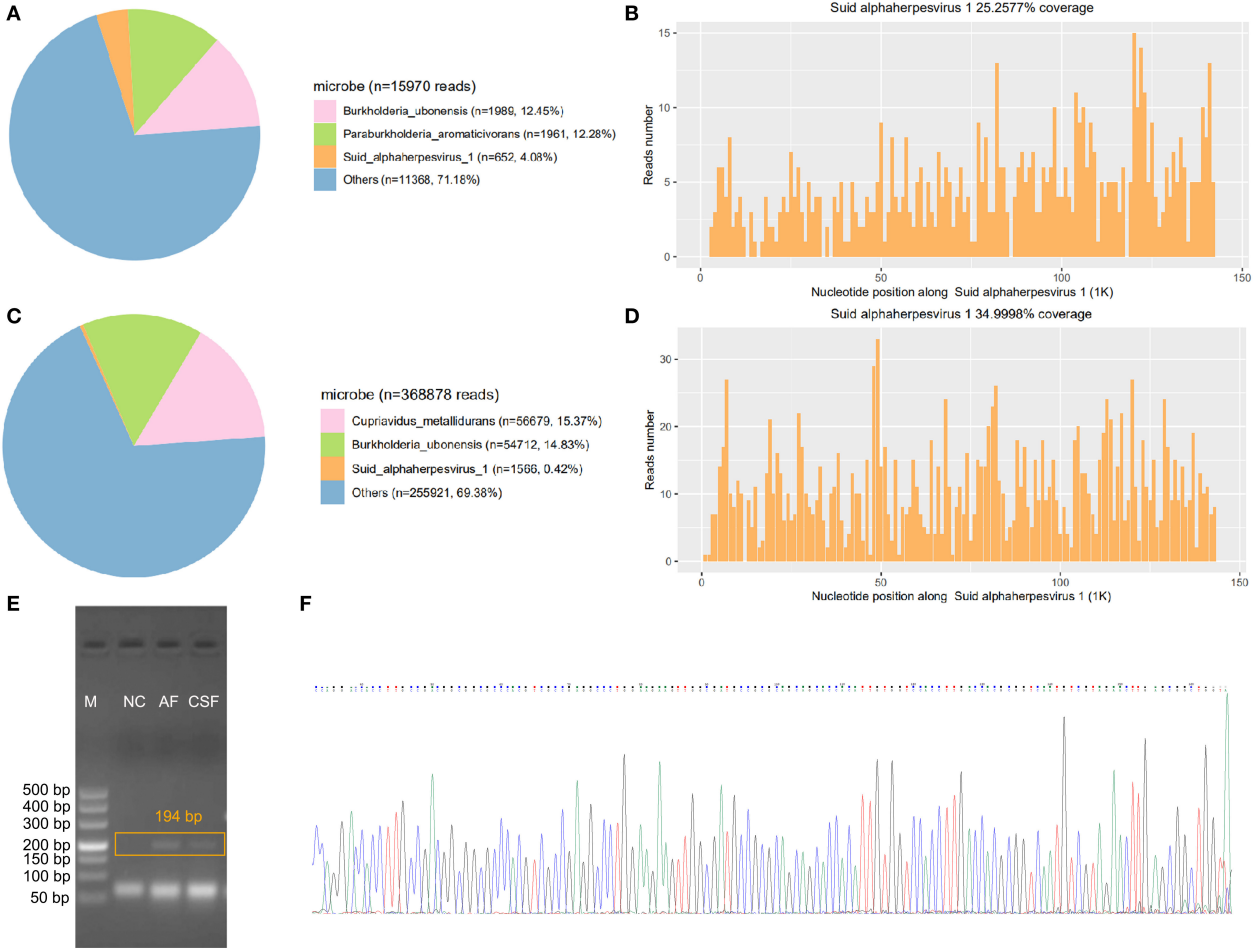
Findings of mNGS and PCR confirmation; **(A,B)** mNGS results of the CSF. **(C,D)** mNGS results of the aqueous humor; **(E)** Gel electrophoresis of PCR products; **(F)** Sanger sequencing result; Genome coverage rate is total length of detected sequences dividing by the length of published genome sequence. The “AH,” “CSF,” “NC,” and “M” represent aqueous humor, cerebrospinal fluid, negative control, and marker, respectively.

A PACEseq mNGS detection (Hugobiotech, Beijing, China) was performed. The QIAamp DNA Micro Kit (QIAGEN) was used to extract DNA. The library was then built using QIAseq™ Ultralow Input Library Kit (Illumina, San Diedo, CA, USA). The library quality was assessed using Qubit (Thermo Fisher) and Agilent 2100 Bioanalyzer (Agilent Technologies, Santa Clara, CA, USA), and was finally sequenced on Nextseq 550 platform (Illumina, San Diedo, CA, USA). Short, low-quality, and low-complexity reads were removed from the raw data. The human DNA was also filtered out by alignment to human reference database (hg38) using bowtie 2. The remaining reads were finally aligned to Microbial Genome Databases (ftp://ftp.ncbi.nlm.nih.gov/genomes/) by BWA. A total of 652 unique reads mapped to the Suid alphaherpesvirus 1 genome out of 15,970 microbial reads (Figure 2A) with a genome coverage rate of 25.3% (Figure 2B) in CSF sample, whereas only one unique read of Suid alphaherpesvirus 1 in blood sample was detected, which might be due to the low concentration of viral DNA in blood. Notably, mNGS of aqueous humor sample detected 1,566 unique reads (0.42%, 1,566/368,878) (Figure 2C) aligned to the Suid alphaherpesvirus 1 genome with a genome coverage rate of 35% (Figure 2D). Furthermore, PCR targeting *glycoprotein B* (*gB*) gene of Suid alphaherpesvirus 1 showed that fragments of about 194 bp in length were successfully amplified from CSF and aqueous humor samples (Figure 2E). The identity of the fragment sequence obtained from a Sanger sequencing, against the reference sequence of Suid alphaherpesvirus 1 (GenBank accession no. MK806387.1) was 99% (Figure 2F). However, a negative PCR result was found in blood sample due to the low viral load. The above results confirmed the presence of Suid alphaherpesvirus 1 in CSF and aqueous humor samples.

## Discussion

Pseudorabies virus (PRV) is an alpha herpesvirus that causes neurological diseases in many wild and domestic animals. The PRV infection is highly prevalent among swine. This virus, which takes swine as its natural host, has been found in humans in 1914, but those cases were debatable because they lacked evidence of antibodies or PRV sequences. Three suspected cases of human PRV infection with positive antibodies were reported in 1987, strongly indicating that PRV could infect human (3). In 2018, Ai et al. reported a case of human endophthalmitis caused by PRV infection, and this is the first case of human PRV endophthalmitis revealed by mNGS and PCR, while PRV was only detected in the aqueous humor in this case (6). Among the reported cases, the main manifestation was severe encephalitis or endophthalmitis, respectively (7–11). While, in our case, PRV, as detected by mNGS in blood, CSF, and aqueous humor samples, exhibited a wider range of infections.

Clinical manifestations and neuroimaging changes of our patient was basically consistent with those of patients with PRV encephalitis as previously reported (7–11). The clinical characteristics of suspected pseudorabies encephalitis (PRE) are as follows: (I) the patients mainly engaged in the production, processing or marketing of live swine and raw pork; (II) acute onset with fever, headache, and other “cold-like symptoms”; (III) severe encephalitis with rapid progress characterized by seizures, disturbance of consciousness, ICU treatment often needed, and even ventilator-assisted breathing; (IV) other systems, such as endophthalmitis; (V) The PRE neuroimaging show necrotizing encephalitis mainly involved gray matter, including limbic system, basal ganglia, and brainstem; (VI) The CSF exhibits characteristics of aseptic meningitis or viral meningitis, including elevated intracranial pressure (ICP), a mild to moderate increase of WBC, lymphocytic inflammation in CSF cytology, elevated CSF protein and so on; (VII) Antiviral drugs, human immunoglobulins and steroid hormones can be tried, but effective antiviral treatments have yet to be determined. In addition to the typical severe cases, there are also cases with relatively mild clinical manifestations or incomplete involvement.

In our case, the early diagnosis was delayed, mainly due to the early “cold-like symptoms,” which did not arouse the attention of the patient and, thus, early effective treatment was not delivered. In the later stage, the clinical manifestations of the patient progressed to the typical manifestations of severe encephalitis. As the patient rapidly progressed to a coma, the endophthalmitis of the patient was not diagnosed. The PRV in the aqueous humor was found positive in the follow-up body fluid testing. Plain CT is not sensitive enough in the early stage of disease, and as the disease progressed, it showed that the characteristic neuroimaging changes, our patient showed hypo-density lesions in the bilateral frontotemporal lobe, basal ganglia, thalamus, and insular lobe. Repeated CSF tests are suggestive of viral meningitis, mainly with elevated ICP and mild increase of WBC. We used mNGS to place a strict alignment of the PRV in the blood, CSF and aqueous humor of the patient, while the nucleic acid for Herpes simplex virus (HSV) and other DNA viruses were negative. Other clinical virology tests and pathogen tests of the patient were negative, as well as the autoimmune encephalitis antibodies, which helped to exclude other causes of encephalitis.

Antiviral drugs should be used for initial treatment of patients with highly suspected viral encephalitis. Postponing antiviral therapy (more than 48 h) can lead to a poor prognosis, including ocular complications-acute retinal necrosis syndrome (12). Acyclovir, as the main drug for the Herpes simplex encephalitis (HSE), may not have an ideal effect on a severe PRV encephalitis. Accordingly, we added phosphonoformate as the intensive antiviral therapy for this patient. Meanwhile, human immunoglobulins and glucocorticoids can also be tried, and we used methylprednisolone sodium succinate for a short period of time, while the condition of the patient had not been effectively improved. Although we successfully detected PRV by using mNGS and a corresponding antiviral therapy was also performed, the prognosis of this patient was worse without timely and accurate diagnosis in the early stage of PRV infection, emphasizing the importance of mNGS in early diagnoses.

The mNGS results of the patient demonstrated the presence of PRV in blood, CSF, and aqueous humor, which was a newly identified virus that infects human. In addition, mNGS data also showed high reads number and coverage rate of PRV, which was further confirmed by PCR and Sanger sequencing, validating the credibility of the mNGS technology. Specific viral diagnosis could be achieved by demonstration of viral nucleic acid, or antibody in CSF or isolation of the virus from CSF or brain tissue (12). The sensitivity of traditional CSF diagnostic methods for CNS viral infections is very low, because the short period of viremia and difficulty in obtaining brain tissue through biopsy make it difficult to isolate viruses. However, the state-of-the-art mNGS technology could detect all the pathogens in the clinical sample without a priori and with high sensitivity. It has been more and more accepted as a rapid and precise method for clinical diagnostics of infectious diseases, including sepsis, meningitis, and pneumonia. The mNGS performs a great advantage in clinical diagnosis, especially for rare, new, and mixed infections (13). There are also some limitations in using mNGS. The key limitation of mNGS is that microbial nucleic acids from the patients' samples are dominated by human host background, which limits the overall analytical sensitivity of the approach for pathogen detection, given the relative scarcity of microbial non-human reads that are sequenced. However, mNGS is a revolutionary technology that has disrupted traditional clinical diagnostics on several fronts.

Since 2017, reported cases of PRV encephalitis or endophthalmitis were all from China. In 2020, hSD-1/2019, a human-originated PRV strain, was isolated from an acute human encephalitis case, and showed close phylogenetic relationships and similar etiological characteristics to Chinese PRV variant strains, implying that there may be certain risks of PRV transmission from swine to human (14). It was hypothesized that the PRV detected in our case may be a variant strain rather than PRV classical strain, which can be further confirmed by a whole genome sequencing. Thus, we went back to the slaughterhouse to collect the residual blood from the slaughter knife for further analysis, but PRV virus was not detected. It may be due to the fact that the knife had been exposed to the air for a long time. The swine slaughtered by the patient had been sold on the market, so we can no longer trace the source of the infection. Additionally, novel Chinese PRV variant strain may lead to failure of the standard Bartha-K61 vaccine to protect swine against the PRV (15). Given the current global epidemic of PRV in swine, this “new life-threatening zoonosis” deserves more attention, and the mechanism of human PRV infection requires further investigation.

## Conclusion

We found that PRV infection can result in prominent central nervous system (CNS) disorders and fatal encephalitis in human. For cases of encephalitis with unknown etiology, if the epidemiology, clinical manifestations, CSF, and brain imaging conform to the PRE, PRV nucleic acid testing and serological testing should be perfected as soon as possible. The mNGS helps to rapidly screen for new viral encephalitis such as PRV from encephalitis of an unknown etiology. The PRE are rare encephalitis which are detectable, treatable, and preventable. People who worked in the animal husbandry and veterinary should increase awareness of self-protection when contacting swine, as broken skin or mucous membrane might make it easy for PRV to transmit from infected animals to human. Establishing accessible diagnostic protocol, determining effective antiviral treatment, exploring the infection pathway and pathogenic mechanism of PRV to human are all the remaining problems to be solved.

## Data Availability Statement

The datasets presented in this article are not readily available due to ethical and privacy restrictions. Requests to access the datasets should be directed to the corresponding author.

## Ethics Statement

The studies involving human participants were reviewed and approved by Medical Professional Committee of the Second Xiangya Hospital of Central South University. The patients/participants provided their written informed consent to participate in this study. Written informed consent was obtained from the individual(s) for the publication of any potentially identifiable images or data included in this article.

## Author Contributions

WY wrote the manuscript. WY, ZH, and YZ helped to collect the clinical data and neuroimages. XW and HZ analyzed the data and made revisions of the manuscript. All authors have read and agreed to the published version of the manuscript.

## Funding

This research was supported by grant of National Natural Science Foundation of China (No. 81801123) to WY.

## Conflict of Interest

The authors declare that the research was conducted in the absence of any commercial or financial relationships that could be construed as a potential conflict of interest.

## Publisher's Note

All claims expressed in this article are solely those of the authors and do not necessarily represent those of their affiliated organizations, or those of the publisher, the editors and the reviewers. Any product that may be evaluated in this article, or claim that may be made by its manufacturer, is not guaranteed or endorsed by the publisher.
