# Supplementary figures and images for: Case Report: Metagenomic Next-Generation Sequencing for Diagnosis of Human Encephalitis and Endophthalmitis Caused by Pseudorabies Virus
Source: Front Med (Lausanne). 2022 Jan 14;8:753988. doi: 10.3389/fmed.2021.753988 (PMC8795075; doi:10.3389/fmed.2021.753988)

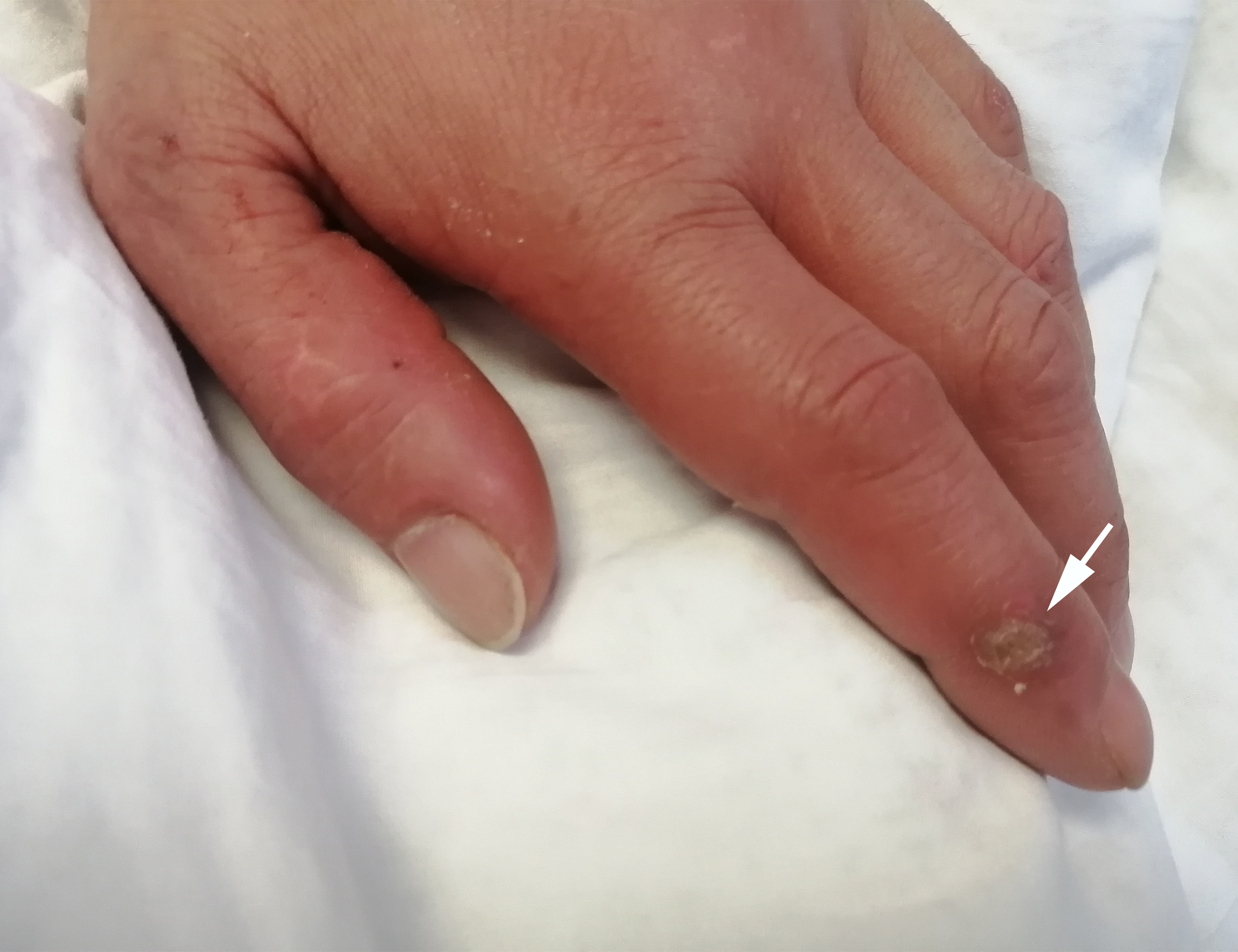

Supplement: Supplementary file 1 [file Image_1.jpg]
